# Editorial: Molecular crosstalk between endocrine factors, paracrine signals, and the immune system during aging

**DOI:** 10.3389/fendo.2023.1203755

**Published:** 2023-04-21

**Authors:** Durai Sellegounder, Luigi Ferrucci, Rajakumar Anbazhagan, Nathan Basisty

**Affiliations:** ^1^ Buck Institute for Research on Aging, Novato, CA, United States; ^2^ Translational Gerontology Branch, National Institute on Aging, National Institutes of Health, Bethesda, MD, United States; ^3^ Eunice Kennedy Shriver National Institute of Child Health and Human Development, National Institutes of Health, Bethesda, MD, United States

**Keywords:** aging, endocrinology, immune system, paracrine signal, metabolism & endocrinology, hormones, senescence

## Introduction

Aging is a complex biological process that gradually declines physiological function and increases susceptibility to disease. Various factors, including genetics, lifestyle, and environmental factors, influence this process (1). One key factor that has emerged as a significant contributor to aging is the interactions between the endocrine, paracrine, and immune systems. The endocrine and immune systems are closely interconnected and work together to maintain homeostasis in the body. Hormones produced by the endocrine system, such as insulin, growth hormone, prolactin, and thyroid hormone, have a significant effect on the immune system. For example, Insulin increases production of inflammatory cytokines like IL-6 during LPS stimulation in macrophages (2). Conversely, immune cells, such as T and B cells, and senescent cells, can produce hormones that regulate immune function and interact with the endocrine system. For example, senescent cells, *via* the senescence-associated secretory phenotype (SASP), release growth factors and cytokines that interact with the local and systemic environment (3, 4). The endocrine and immune systems utilize paracrine signaling to coordinate cellular responses to any stress in distant tissues in the body. There is a significant gap in the field regarding our understanding of the interaction between these pathways during aging and diseased conditions.

The current Research Topic, “*Molecular Crosstalk Between Endocrine Factors, Paracrine Signals, and the Immune System During Aging*,” helps to bridge this gap by highlighting recent research findings at the intersection of endocrine-immune axis, aging, and age-related pathologies, as well as opportunities for therapeutic interventions.

## Highlights of manuscripts in this research topic

Endocrine factors regulate many physiological processes, including growth and development, metabolism, and reproduction. With age, the levels of many endocrine factors change, leading to alterations in these processes. For example, insulin receptor (InsR) signaling is a well-conserved pathway regulating longevity. Makhijani et al. reviewed the function of InsR signaling pathways in different immune cell subsets and their impact on cellular metabolism, differentiation, and effector versus regulatory function. With ample evidence from the literature, the authors provided mechanistic links between altered InsR signaling and immune dysfunction in various disease settings and conditions, focusing on age-related conditions, such as type 2 diabetes and cancer.

The immune system plays a key role in defending the body against infection and disease. As we age, the immune system undergoes significant changes, including a decline in the production of new immune cells and a decrease in the ability of immune cells to respond to infection. The reduction in immune cells can lead to an increased susceptibility to infections and a reduced ability to clear infections once they occur. King et al. provided a brief report on the relationship between aging, reproductive health, and immune function. From the studies in the lab, the authors claim that transplanting young ovaries into old mice increased healthspan and lifespan. However, the results from Mason’s lab suggest that the protective effect of the ovarian transplant was not due to hormonal activity, as hormone-depleted ovaries from young mice also extended their lifespan. The authors claim that additional factors other than ovarian hormones are the reason for health benefits. In their current report, the authors specifically focused on the influence of young ovarian tissues on immune function in post-reproductive female mice in the presence or absence of ovarian follicles.

Hormones play an essential role in the immune system and can significantly impact the development and progression of rheumatic disorders. Bertoldo et al. reviewed the interaction between the endocrine hormones and the immune system from the perspective of rheumatic disorders. The review article covers recent data describing the role of bone-related hormones and cytokines.

The pituitary gland produces growth hormone (GH), which plays a key role in growth and development during childhood and adolescence. While some studies have suggested that GH replacement therapy may improve markers of health and longevity in older adults, other studies have raised concerns about GH treatment’s potential risks and side effects, such as an increased risk of cancer and diabetes. As a part of this Research Topic, Bartke reviewed the relationship between growth hormones and longevity. He suggested that a slower pace of life is associated with extended longevity within and between species. This review warrants future studies in understanding energy metabolism and nutrient-dependent signaling at different stages of life.

Paracrine signals are molecules produced by one cell and act on neighboring cells to regulate their function. These signals are vital in maintaining tissue homeostasis and responding to damage or injury. In aging, the production and response to paracrine signals can become dysregulated, leading to tissue dysfunction and disease. For example, senescent cells’ production of inflammatory cytokines can lead to chronic inflammation, a hallmark of aging, and associated with many age-related diseases. Kuehnemann et al. reported a new senescence-associated secretory phenotype marker. Nicotinamide Phosphoribosyl Transferase (NAMPT), the enzyme involved in the rate-limiting step of NAD biosynthesis, is increased in senescent cells. Results from the research show that the senescence cells displayed increased NAMPT, which is different from classical DNA damage response and without further increase in NAD. Based on the observed results, the authors believe that increased extracellular NAMPT (eNAMPT) during senescence is another SASP marker that could regulate metabolic functions in distant cells. Further, the authors showed that diabetic mice displayed elevated levels of eNAMPT, and treatment with the senolytic drug ABT-263 can rescue the high levels of eNAMPT.

## Conclusion and future perspective

In conclusion, the crosstalk between endocrine factors, paracrine signals, and the immune system is a complex and dynamic process that plays a crucial role in aging. The interaction between these vital pathways has important implications for aging research and interventions. For example, targeting endocrine factors such as growth hormone and IGF-1 or paracrine signals such as inflammatory cytokines may provide new therapeutic strategies to improve immune function in the elderly population. The review articles and research manuscripts presented in this Research Topic have highlighted this crosstalk’s importance and identified new intervention targets. Further research is needed to fully understand the dynamic interactions between biological pathways and develop effective interventions to improve health and prevent age-related diseases.

## Author contributions

All authors listed have made a substantial, direct, and intellectual contribution to the work and approved it for publication.
